# Recent insight into the role of macrophage in alcohol-associated liver disease: a mini-review

**DOI:** 10.3389/fcell.2023.1292016

**Published:** 2023-11-29

**Authors:** Jialiang Sun, Peiliang Zhao, Ying Shi, Yanan Li

**Affiliations:** ^1^ Department of Pediatrics, The First Hospital of Jilin University, Changchun, China; ^2^ Department of Hepatology, The First Hospital of Jilin University, Changchun, China

**Keywords:** alcohol-associated liver disease, kupffer cells, macrophage, macrophage polarization, macrophage phenotypes

## Abstract

Alcohol-associated liver disease (ALD) is a condition that develops due to prolonged and excessive alcohol consumption. It encompasses various stages of liver damage, including fatty liver, alcoholic hepatitis, and cirrhosis. Immune cells, particularly macrophages, of various types play a significant role in the onset and progression of the disease. Macrophages observed in the liver exhibit diverse differentiation forms, and perform a range of functions. Beyond M1 and M2 macrophages, human macrophages can polarize into distinct phenotypes in response to various stimuli. Recent advancements have improved our understanding of macrophage diversity and their role in the progression of ALD. This mini-review provides a concise overview of the latest findings on the role and differentiation of macrophages in ALD. Additionally, it discusses potential therapeutic targets associated with macrophages and explores potential therapeutic strategies.

## Introduction

Alcohol-associated liver disease (ALD), a group of liver diseases triggered by prolonged excessive alcohol consumption, is a leading cause of chronic liver disease and liver transplantation globally. It encompasses various stages, including fatty liver, alcoholic hepatitis, and cirrhosis (Singal et al., 2018). The immune system plays a critical role in the progression and pathogenesis of ALD.

The liver, a hub of metabolism, biotransformation, and immune regulation, is a site of complex immune activities mediated by diverse immune cell pools and non-hematopoietic cell populations. Consequently, the liver, housing various types of innate and adaptive immune cells including natural killer (NK) cells, Kupffer cells (KCs), macrophages, and more, is deemed an immune organ. These cells are integral in maintaining immune homeostasis, capable of maintaining immune tolerance and resisting pathogens (Chen and Tian, 2021). In ALD, alcohol-induced liver injury disrupts the delicate balance between immune tolerance and activation. The altered liver microenvironment, coupled with the presence of alcohol metabolites, can modulate immune cell development and function, consequently leading to disease progression (Byun and Yi, 2017). Macrophages, essential components of the innate immune system, are key players in the pathogenesis of ALD, being associated with liver injury, inflammation, and fibrosis. Macrophages interact with alcohol metabolites, gut-derived bacterial products, and cytokines. The macrophages observed in the liver after injury are heterogeneous and may originate from different sources, the origin of the cells is associated with variations in cell function and responsiveness to activation and recruitment signals, thereby directly influencing the outcome of the immune response (Dou et al., 2019).

This mini-review aims to shed light on the current understanding of the role of macrophages in ALD, including the polarization, the macrophage and other hepatic cells crosstalk in the progression of ALD, and associated potential therapeutic targets.

## The role of macrophages in the pathogenesis of alcohol-associated liver disease

In ALD, identifying pathogen-associated molecular patterns (PAMPs) and damage-associated molecular patterns (DAMPs) is a critical step in the pathogenesis of ALD. Identifying PAMPs and DAMPs is a critical step in the pathogenesis of ALD. PAMPs derived from intestinal microbiota and DAMPs released from damaged cells are recognized by different Toll-like receptors (TLRs) and NOD-like receptors (NLRs) expressed by immune cells and liver parenchymal cells, responding through a combination of pro- and anti-inflammatory signals within the tissue, which helps to remove pathogens, dead cells, and reduces inflammation. However, under chronic alcohol exposure, the recognition of PAMPs and DAMPs may contribute to the progression of alcohol-associated liver inflammation (Dhanda and Collins, 2015). ALD progresses through various clinical stages. In the early stage of ALD, alcohol affects the liver through the hepato-intestinal axis, that is, the two-way communication between the intestine and the liver: chronic alcohol consumption can induce structural alterations in the intestinal epithelial cells of both humans and mice, thereby augmenting intestinal sensitivity. The permeability of macromolecules will lead to leakage of lipopolysaccharides (LPS) originating from the intestine and increase LPS in the portal circulation. As reviewed by Li et al., 2019, intestinal-derived LPS, activate nuclear factor kappa B (NF-κB) and further activate KCs through TLRs, resulting in pro-inflammatory cytokines such as IL-6, TNF-α, and IL-1β, and the production of chemokines such as CC chemokine ligand 2 (CCL2), CCL20, and monocyte chemoattractant protein-1 (MCP-1), which are recruited to the liver, contributing to the early onset of alcoholic hepatic steatosis and the inflammatory response. Subsequent alcohol abuse leads to the development of alcoholic hepatitis and fibrosis. At this stage, cellular dysfunction and programmed cell death stimulate hepatic stellate cells, leading to the production of extracellular matrix (ECM) proteins and potentially resulting in fibrosis or cirrhosis (Koda et al., 2021). Dynamic shifts in macrophage polarization significantly influence different stages of ALD. Certain signals, including pro-inflammatory cytokines (such as IL-6, interferon (IFN)-γ, and TNF-α) and anti-inflammatory cytokines (like IL-10), activate macrophage polarization. This leads to the generation of distinct phenotypes and regulates multiple signaling pathways. Additionally, the pathogenesis of ALD entails intricate interactions between macrophages and other liver cell types such as neutrophils, natural killer (NK) cells, and hepatic stellate cells (HSC). Consequently, macrophages, as integral components of the innate immune system, have been identified as pivotal actors in propagating the inflammatory response and tissue damage in ALD.

## The mechanism of macrophage polarization in alcohol-associated liver disease

Traditionally, macrophages are classified into two principal types during the inflammatory response, “M1” and “M2,” based on their functional attributes. Pro-inflammatory properties are exhibited by classically activated “M1” macrophages, whereas alternatively activated “M2” macrophages display anti-inflammatory and pro-fibrotic characteristics (Liu et al., 2023). It is now recognized that dynamic shifts in macrophage polarization act as critical determinants of ALD pathogenesis. Elucidating the molecular pathways governing macrophage polarization provides insight into potential therapeutic targets for this disease.

Numerous studies have now outlined the key molecular mechanisms that mediate macrophage polarization in ALD. In the early stages of ALD, the liver microenvironment is characterized by the release of DAMPs and PAMPs, promoting macrophages to adopt a pro-inflammatory M1-like phenotype. A principal pathway is the TLR4/NF-κB pathway, driven by gut-derived PAMPs like LPS. LPS binding to TLR4 on KCs triggers the activation of NF-κB and mitogen-activated protein kinase (MAPK) signaling cascades, driving the transcription of pro-inflammatory genes characteristic of M1 polarization (Fang et al., 2022). The production of inflammatory cytokines (like TNF-α, IL-1β, and IL-6) and chemokines (such as CC chemokine ligand 2 (CCL2), CCL20, and MCP-1) further establishes an inflammatory microenvironment, creating a self-perpetuating loop that perpetuates M1 activation. Emerging research has highlighted the role of microRNAs (miRNAs) on macrophage polarization, mainly relying on their regulation of other signaling pathways. For example, chronic alcohol exposure reprograms the responses of KCs to other TLR ligands through the upregulation of miRNAs (like miR-155, miR-125a-5p) and epigenetic regulators, amplifying NF-κB activity and M1 polarization (Wang et al., 2018; Kim et al., 2019). Furthermore, oxidative stress and mitochondrial dysfunction, induced by alcohol metabolism, serve as additional stimuli favoring M1 polarization. While acute M1 activation is protective, aiding in the removal of pathogens and necrotic cells, chronic M1 stimulation initiates and amplifies liver inflammation seen early in ALD, such as alcoholic steatosis and alcoholic hepatitis (Kim et al., 2019). Additionally, in the context of ALD, the Janus kinase (JAK)/signal transducer and activator of transcription (STAT) signaling pathway, a critical regulator of macrophage polarization, is activated by alcohol. This promotes the activation of JAK2 and STAT1, leading to the transcription of M1-associated genes, including those encoding TNF-α and IL-6 (Pan et al., 2021). In summary, alcohol creates a microenvironment that polarizes resident macrophages towards an M1 phenotype in the early stages of ALD.

Conversely, the resolution of inflammation necessitates a phenotypic switch towards M2 macrophages. As ALD progresses, the liver microenvironment changes, prompting macrophages to adopt an M2-like anti-inflammatory phenotype. M2 macrophages play a role in tissue repair and wound healing, some cytokines such as IL-6 play dual functions in ALD. IL-10 signaling, by suppressing NF-κB activity and TLR4 responses, plays a pivotal role in this process (Hakeem et al., 2023). IL-6, derived from hepatocytes, has also been identified as a significant stimulus driving the transition from M1 to M2 (Zhang et al., 2023). STAT3, a downstream target of IL-10 and IL-6, becomes activated and translocates to the nucleus, where it induces the expression of M2 genes (Hakeem et al., 2023; Zhang et al., 2023). Other sources of cytokines like IL-4, IL-13, and transforming growth factor-β (TGF-β) further reinforce M2 polarization through the activation of STAT6 (Guo et al., 2023). TGF-β regulates the expression of related genes by activating its downstream signaling molecules, Smad2 and Smad3. This promotes the phosphorylation of Smad2 and Smad3, inhibiting M1 and promoting M2 polarization. Studies have demonstrated that the TGF-β/Smads signaling pathway inhibits M1 polarization and promotes M2 polarization. Role in promoting M2 polarization. The phosphatidylinositol-4,5-bisphosphate 3-kinase (PI3K)/protein kinase B (Akt) signaling pathway, which plays a crucial role in regulating macrophage polarization and the balance between pro-inflammatory M1 and anti-inflammatory M2 states, has recently become a research focus in chronic liver diseases due to its role in promoting M2 polarization (Yang et al., 2023). Chronic exposure to alcohol leads to increased activation of the PI3K/Akt signaling pathway in macrophages (Cheng et al., 2022; Mehra et al., 2022). Reports indicate that the PI3K/Akt1 pathway upregulates IL-1 receptor-associated kinase M and suppresses TLR4/NF-κB signaling by deactivating tumor necrosis factor receptor-associated factor 6. This downregulates pro-inflammatory cytokine production and contributes to the skew towards M2 polarization (Vergadi et al., 2017). However, in the context of chronic alcohol consumption, a persistent inflammatory state and inadequate resolution can lead to progressive liver injury; prolonged activation of M2 macrophages can contribute to pathological fibrosis, a hallmark of advanced ALD (Wang et al., 2021).

## Beyond the M1/M2 dichotomy: macrophage heterogeneity in alcohol-associated liver disease

Beyond M1 and M2 macrophages, human macrophages can polarize into distinct phenotypes in response to various stimuli. Therefore, the functional classification of macrophages into M1 and M2 is generally considered an oversimplification of the diverse activation states of macrophages (Murray and Wynn, 2011). The M2 category is believed to be further subdivided into M2a, M2b, M2c, and M2d subtypes, each triggered by a different stimulus and exhibiting a unique response. M2a macrophages, for instance, are induced by IL-4 and IL-13. Alternatively activated macrophages, primarily involved in wound healing and tissue repair, constitute one subtype. M2b macrophages, triggered by immune complexes and/or LPS, hold both protective and pathogenic effects. M2c macrophages, stimulated by IL-10, TGF-β, and glucocorticoids, exhibit regulatory phenotypes and play roles in suppressing inflammation, and fibrosis, and promoting tissue repair. M2d macrophages, activated by IL-6, TLR ligands, and adenosine, share functional attributes with tumor-associated macrophages (TAMs). Collectively, these M2 macrophages dampen immune responses and foster tissue repair (Shrivastava and Shukla, 2019). On the other hand, several variants of macrophage immunophenotyping have been reported. M1 and M2 macrophages produce cytokines and oxidized phospholipids that foster the development of macrophages into metabolically activated macrophages (MMe) and oxidative macrophage (Mox) populations (Skuratovskaia et al., 2020). This classification proves useful in comprehensively describing the function of macrophages, for instance, in obese individuals. The functional classification method is premised on the differential expression levels of cluster of differentiation (CD)14 and CD16 molecules on the cell surface. This classification framework distinguishes three major groups: classical (CD14^high^CD16^−^), intermediate (CD14^high^CD16^low^), and non-classical macrophages (Ziegler-Heitbrock, 2015). Moreover, Kalish et al., 2017 proposed a hypothesis that macrophages could facilitate interconversion between M1 and M2 states by forming an M3 switching phenotype which was observed in a mouse model of abdominal tumors. In 2015, Erbel et al., 2015 demonstrated that CXCL4 could induce a macrophage phenotype, termed M4, characterized by the co-expression of CD68, matrix metalloproteinase (MMP)-7, and calcium-binding protein S100A8.

Recent advances in single-cell RNA sequencing (scRNA-seq) have provided insights into the diversity of liver macrophages and their regulation during ALD progression, thereby deepening our molecular understanding of this disease, and extending beyond the traditional M1 and M2 macrophage paradigm (Zhang et al., 2021). A recent study, using scRNA-seq data from the human liver, identified two subpopulations of macrophages. Furthermore, MARCO (Macrophage Receptor with Collagenous structure) is exclusively expressed in non-inflammatory KCs and serves as a marker to differentiate between different macrophage populations in the human liver (MacParland et al., 2018). *In vitro*, the researchers stimulated these two macrophage populations and assessed their cytokine secretion. They observed that *Marco* ^*positive(+)*^macrophages showed decreased TNF-α secretion in response to LPS/IFN-γ stimulation compared to *Marco* ^*negative(−)*^ *CD68* ^*+*^ macrophages, suggesting that *CD68* ^*+*^ *Marco* ^*-*^ cells possess more pro-inflammatory properties (MacParland et al., 2018). Tumor-associated macrophages (TAMs) are a diverse population of myeloid cells found in the tumor microenvironment. They play a role in immune suppression and facilitate the initiation, growth, and spread of solid tumors. In mouse models of colon cancer, the expression of Marco was found to identify a specific subset of suppressive TAMs. The expression of Marco on immunosuppressive M2 TAMs is associated with both immunosuppressive and tumor-promoting effects. Anti-Marco antibodies polarized it to a pro-inflammatory phenotype and enhanced tumor immunogenicity by reprogramming the TAM populations (Georgoudaki et al., 2016). Therefore, characterizing macrophage populations provides a valuable framework to investigate the roles of macrophage subsets in liver disease and identify unique functional pathways of distinct intrahepatic monocyte/macrophage populations.

## Crosstalk between macrophage and other hepatic cells in alcohol-associated liver disease

Hepatocytes, as the primary liver cells, are the main targets of damage induced by ethanol. Hepatocyte apoptosis is led by chronic alcohol consumption. A principal pathway underlying ALD involves the endotoxin-mediated activation of KCs, triggering the release of inflammatory mediators such as TNF-α and IL-6. These mediators can engage neighboring hepatocytes and exacerbate inflammation (Wang et al., 2021). TNF-α synergizes with LPS, sensitizing hepatocytes to apoptosis through the stimulation of the JNK/p38 MAPK pathway (Choi et al., 2023). Furthermore, IL-6 derived from KCs enhances the recruitment of neutrophils in the liver and contributes to tissue damage, chronic alcohol exposure increases LPS-induced neutrophil extracellular trap (NET) production, polarizing macrophage towards M1 phenotype (Cho et al., 2023). In ALD, cytokines such as IL-12, IL-18, and TNF-α, derived from KCs, promote the activation of NK cells. This enhances their cytotoxic function and their production of IFN-γ, a critical cytokine for controlling liver inflammation and fibrosis.

**FIGURE 1:**
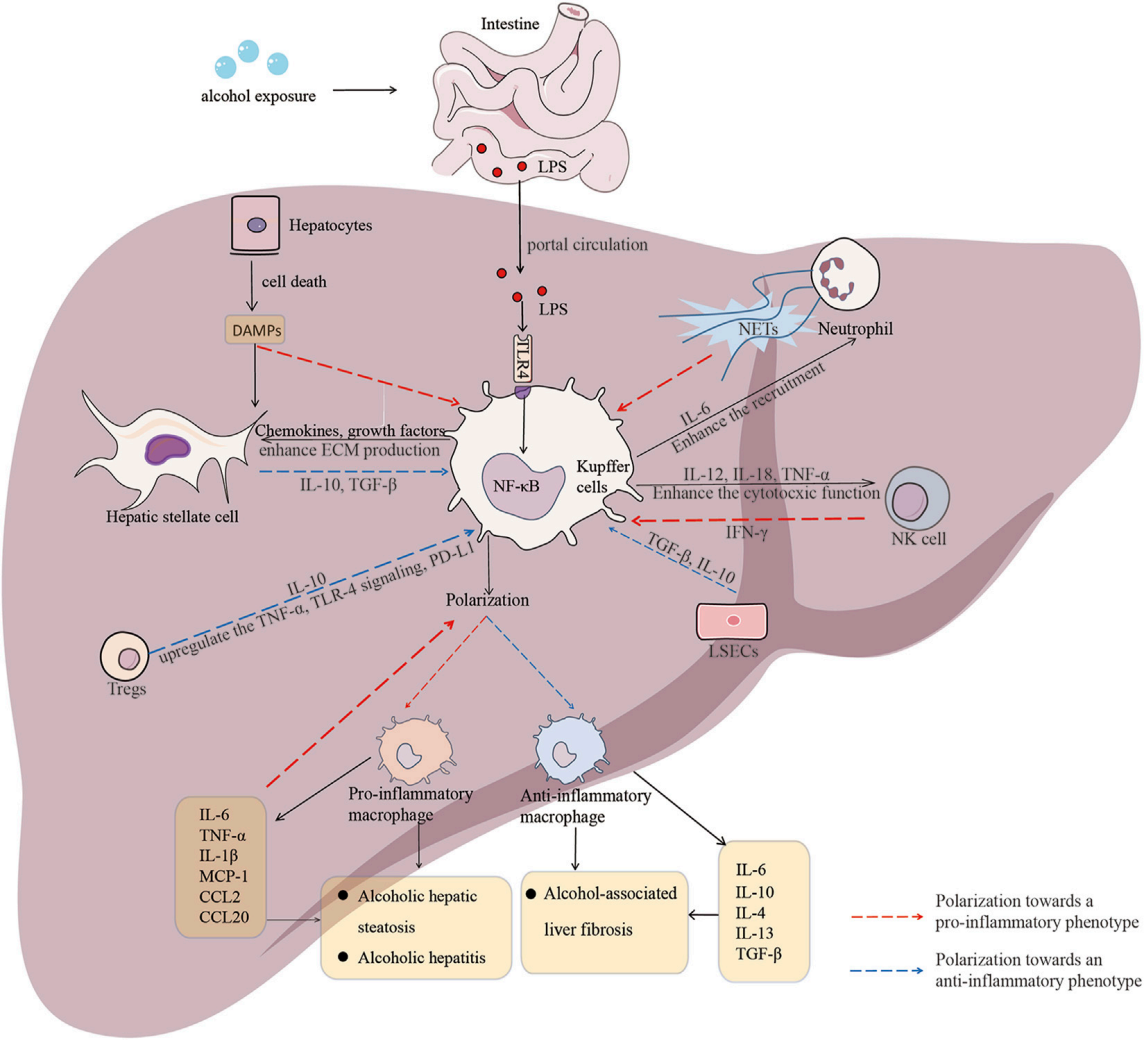
Macrophage polarization and crosstalk between macrophage and other hepatic cells in the context of ALD. Dynamic shifts in macrophage polarization significantly influence different stages of alcohol-associated liver disease (ALD). Various signals, including pro-inflammatory cytokines and chemokines, as well as anti-inflammatory cytokines, initiate macrophage polarization, resulting in the generation of distinct phenotypes and the regulation of multiple signaling pathways. Additionally, the pathogenesis of ALD involves intricate interactions and mutual feedback between macrophages and other hepatic cell types, such as neutrophils, natural killer (NK) cells, and hepatic stellate cells (HSCs). The crosstalk between macrophages and other hepatic cells plays a significant role in either promoting or containing the progression of ALD at different stages.

The pro-fibrotic properties of M2 macrophages significantly contribute to liver fibrosis in ALD. During chronic ethanol exposure, many factors in the liver microenvironment promote HSC activation, release anti-inflammatory cytokines such as IL-10 and TGF-β, polarizing macrophages toward M2 phenotype. The subsequent production of ECM proteins leads to the progressive replacement of liver parenchyma by nonfunctional scar tissue (Matsuda and Seki, 2020). This exemplifies how the crosstalk between KCs and HSCs drives the progressive fibrosis that accompanies alcohol-induced liver inflammation. Emerging studies reveal critical interactions between KCs and LSECs in the pathogenesis of ALD. LSCEs, specialized endothelial cells, play a role in hepatic homeostasis and microcirculation regulation. They allow the exchange of nutrients and waste between hepatocytes and the circulatory system (Gracia-Sancho et al., 2019). LSECs regulate macrophage phenotypes through the paracrine signaling of IL-10 and TGF-β (Casey et al., 2022). Ablation of IL-10 derived from LSECs exacerbates alcohol-induced liver injury, suggesting that LSECs normally suppress the activation of inflammatory KCs. LSECs also constitute a crucial component of the immunosuppressive hepatic microenvironment that dampens the responses of KCs to gut-derived toxins. Regulatory T cells (Tregs) represent another source of anti-inflammatory signals that counterbalance the M1 macrophage response. This is achieved through the production of IL-10 and through direct cell-to-cell contact mechanisms. Additionally, Tregs suppress the production of TNF-α and TLR4 signaling in macrophages. Tregs also upregulate the expression of programmed cell death ligand 1 (PD-L1) on myeloid cells, including KCs, inhibiting their stimulation through PD-1 interactions (Markwick et al., 2015). In this way, adaptive immune cells, such as Tregs and infiltrating monocytes, engage in multicellular signaling that shapes the responses of macrophages in ALD, shown in Figure 1.

## Therapeutic implications: Targeting macrophages in alcohol-associated liver disease

Given the dual pro-inflammatory and anti-inflammatory roles of macrophage polarization in ALD, modulating macrophage polarization may offer a strategy to balance inflammation and repair in ALD [26]. Emerging research has identified various mediators that regulate macrophage activation and polarization. For instance, berberine competitively inhibits TLR4, and blocks the TLR4/MyD88/NF-kB signaling pathway, thereby suppressing M1 macrophage polarization (Gong et al., 2019). Asaronic acid attenuates M1 polarization by inhibiting both the NF-κB signaling and JAK/STAT signaling pathway (Oh et al., 2019). Purinergic receptor P2X, ligand-gated ion channel, 4 (P2X4), a member of purinoceptor family, plays a significant role in alcohol-associated liver fibrosis (ALF). Blockage of the P2X4 purinergic receptor attenuates ALF by enhancing murine M2 macrophage polarization and inhibiting HSC activation via the PI3K/AKT pathway (Li et al., 2022). Recently, probiotics, metformin, and their combination can promote M2 polarization and inhibit M1 polarization, potentially alleviating alcohol-induced liver injury (Patel et al., 2021). Furthermore, elafibranor, a peroxisome proliferator-activated receptors (PPARs)-α/δ dual agonist, has been found to reduce liver steatosis *in vitro*. It achieves this by promoting M2 polarization, inhibiting M1 polarization, and modulating the TLR4/IFN-γ and IL-10/STAT3 signaling pathways (Hakeem et al., 2023). Recent research has also revealed that miR-34a activates M1 polarization and inhibits M2 polarization through the regulation of Recombinant Sirtuin 1 (Sirt1) (Wan et al., 2023). Artificial induction of macrophage polarization is gaining attention as an emerging cell therapy. This approach involves the use of epigenetics and cell survival mechanisms to influence macrophage development and survival, the manipulation of the normal tissue microenvironment, and the use of exogenous factors such as inflammation-induced cytokine release. In recent years, extracellular vesicles (EVs), small membrane-bound vesicles secreted by cells, are capable of transferring biological materials like proteins and miRNAs. They serve as significant effectors of intercellular communication and have been demonstrated to modulate the phenotype of KCs in ALD. For example, EVs carrying heat shock protein 90 have been found to enhance M1 polarization and inhibit M2 polarization, in addition, miR-27, derived from monocyte-derived EVs exposed to alcohol, has been found to induce naive monocytes to differentiate into M2 macrophages (Saha et al., 2018). Thus, despite most of the current research being conducted at the cellular and animal levels, these approaches have demonstrated promising potential in modulating macrophage plasticity, offering promise for macrophage-targeted therapies, as shown in Table 1.

**TABLE 1:** Therapeutic approaches targeting macrophage polarization in ALD.

Regulation factors	Molecular mechanisms	Impacts on macrophage polarization	Research objects	Reference
Berberine	TLR4/MyD88/NF-κB signalling pathway ↓	M1 ↓	Mice and cells	Gong et al. (2019)
Asaronic acid	NF-κB signal ↓; JAK/STAT signaling pathway ↓	M1 ↓	Cells	Oh et al. (2019)
5-BDBD (a P2X4 receptor)	PI3K/AKT signaling pathway ↑	M2 ↑	Mice and cells	Li et al. (2022)
Metformin and Probiotics	LPS/TLR4/NF-κB signaling pathway ↓; MAPK/Nrf-2/HO-1 signaling pathway ↑	M1 ↓; M2 ↑	Rats and cells	Patel et al. (2021)
Elafibranor (a PPARs-α/δ dual agonist)	TLR4/IFN-γ signaling pathway ↓; IL-10/STAT3 signaling pathway ↑	M1 ↓; M2 ↑	Mice and cells	Hakeem et al. (2023)
miR-34a	SIRT1 ↓; p53 ↑	M1 ↑; M2 ↓	Patient samples and cells	Wan et al. (2023)
EV-Hsp90	TLR4/NF-κB signaling pathway ↑	M1 ↑	Mice and cells	Saha et al. (2018)

Abbrevations: ↓: inhibit; ↑: promote; TLR: Toll-like receptors; MyD88: myeloid differentiation factor 88; NF-κB: nuclear factor kappa B; JAK: janus kinase; STAT: signal transducer and activator of the transcription; 5-BDBD:5-(3-Bromophenyl)-1,3-dihydro-2H-benzofuro [3,2-e]-1,4-diazepin-2-one; PI3K: phosphatidylinositol-4, 5-bisphosphate 3-kinase; Akt: protein kinase B; LPS: lipopolysaccharides; MAPK: mitogen-activated protein kinase; Nrf-2: nuclear factor erythroid 2-related factor 2; HO-1: Hemeoxygenase-1; PPARs: peroxisome proliferator-activated receptors; IFN: interferon; miR: microRNA; SIRT1: Recombinant Sirtuin 1; EV: extracellular vesicles; Hsp90: heat shock protein 90.

## Future perspectives

While the therapeutic targeting of macrophages in ALD holds promise, several challenges persist. These encompass the need for superior drugs with minimal side effects, enhanced targeting of liver macrophages, and the identification of biomarkers to monitor treatment response. Furthermore, considering the heterogeneity of macrophages and their diverse functions in ALD, the timing, and context of treatment become crucial. For instance, although inhibiting macrophage activation might be beneficial in preventing inflammation in early ALD, promoting macrophage activation in later stages could be necessary to enhance liver repair. Nevertheless, care must be taken to avoid macrophage hyperactivation that could promote pathological fibrosis.

In summary, it is necessary to gain a deeper understanding of the complex roles and regulation of macrophages in ALD. With the advancement of technologies such as single-cell RNA sequencing, metabolomics, and bioinformatics, future research is needed to further unravel the complexities of macrophage biology in ALD and to translate these insights into effective patient therapies.
